# Clinical review: The suggested management pathway for urticaria in primary care

**DOI:** 10.1002/clt2.12195

**Published:** 2022-10-05

**Authors:** Dermot Ryan, Luciana K. Tanno, Elizabeth Angier, Evangéline Clark, David Price, Torsten Zuberbier, Marcus Maurer

**Affiliations:** ^1^ Usher Institute University of Edinburgh Medical School Edinburgh UK; ^2^ Department of Allergy University Hospital of Montpellier Montpellier France; ^3^ Institut Desbrest d’Epidémiologie et de Santé Publique IDESP University of Montpellier – INSERM Montpellier France; ^4^ WHO Collaborating Centre on Scientific Classification Support Montpellier France; ^5^ Primary Care, Population Science and Medical Education, Faculty of Medicine University of Southampton Southampton UK; ^6^ Department of Dermatology Montpellier University Hospital Montpellier France; ^7^ Observational and Pragmatic Research Institute Singapore Singapore; ^8^ Centre of Academic Primary Care, Division of Applied Health Sciences University of Aberdeen Aberdeen UK; ^9^ Institute of Allergology Charité – Universitätsmedizin Berlin Freie Universität Berlin and Humboldt‐Universität zu Berlin Berlin Germany; ^10^ Fraunhofer Institute for Translational Medicine and Pharmacology ITMP Allergology and Immunology Berlin Germany

**Keywords:** acute urticaria, chronic urticaria, primary care, urticaria management

## Abstract

**Background:**

Urticaria is a common condition presenting both as acute and chronic disease within primary care. To those without specialist training it is poorly understood from the points of view of diagnosis and management. It causes a considerable disease burden to sufferers with marked impact on quality of life.

**Purpose of this review:**

The recent publication of the EAACI/GA²LEN/EuroGuiDerm/APAAACI Guideline for the Definition, Classification, Diagnosis and Management of Urticaria guideline prompted us to take this excellent resource and re‐configure its findings and recommendations to a non‐specialist audience with particular reference to the needs of the primary care team.

## 1 INTRODUCTION

Skin diseases are the fourth leading cause of non‐fatal morbidity worldwide^1^ with urticaria accounting for 0.45% of all years lost to disability (YLD).^2^

Skin conditions are the most common new reason people present to general practitioners (GPs), a discipline in which many GPs have not had appropriate training; in England and Wales they account for approximately 8.4% of all consultations,^3^ whereas in Spain 5.4% of consultations are mainly for a dermatological problem.^4^

## 2 PREVALENCE OF URTICARIA

Urticaria, has a life‐time prevalence of approximately 20%. The reported prevalence of Chronic Spontaneous Urticaria (CSU) varies from study to study, with a recorded prevalence 0.6% in Spain^5^ to a lifetime prevalence of 1.8% in Germany and a point prevalence of 0.8%.^6^ Females are affected more than twice as frequently as males.^7^, ^8^, ^9^

Age prevalence differs in subtypes, for example, cholinergic urticaria has a peak prevalence between 15 and 30 years of age, CSU around 40 years of age.

## 3 THE RELEVANCE OF THIS TO PRIMARY CARE (PC) AND EMERGENCY DEPARTMENTS (ED)

PC clinicians recognise that there is a deficiency in their dermatology training at all career levels.^10^ More specifically, a recent survey of self‐assessed levels of knowledge and learning needs demonstrated that nearly two thirds of general practitioners (64.8%) professed inadequate knowledge, and nearly three quarters (74.9%) had an urgent learning need with regards to urticaria.^11^ This is reflected in and overlaps with GPs learning needs of acute presentations of food allergy.^12^

Although the GP is considered the first port of call for patients suffering from acute urticaria, there is evidence to suggest that frequently the ED is the initial point of contact, reflecting the urgent desire of patients to attain a diagnosis and treatment in what is essentially a non‐emergency condition. A study from the UK suggested that presentations with urticaria to primary care were more or less static whereas hospital attendances were increasing, perhaps as a consequence of patients using ED as first port of call,^13^ a trend which seems to be also demonstrated elsewhere.^14^

A detailed analysis of admissions performed in Spain demonstrated that urticaria accounted for 8.7% of attendances at the ED.^15^ A further prospective study from Spain showed that approximately 10% of presentations to the Emergency Department are for dermatological complaints and that 12% of these were for urticaria.^16^

## 4 BURDEN OF DISEASE

Many patients with urticaria are inappropriately referred to allergy departments in the belief, by both patient and physician that those patients are suffering from food allergy,^17^ thereby receiving inadequate or inappropriate treatment.^18^ There is a substantial delay in receiving a diagnosis for those suffering from chronic urticaria accompanied by significant morbidity^7^ ^,^ ^19^: Thus, chronic urticaria impacts substantially on the patient in terms of impaired quality of life,^20^ impaired sexual function^21^ and depression^22^ incurring high health care costs as well as high societal costs due to absenteeism and presenteeism.^23^

The recently published 2021 update and revision of the EAACI/GA^2^LEN/EuroGuiDerm/APAAACI guideline for urticaria provides authoritative recommendations for the management of urticaria and has implications for primary care.^24^ This guideline is continuously evolving, giving clear definitions and indications of clinical management as our understanding of the disease develops.

## 5 PURPOSE OF THIS CLINICAL REVIEW

The purpose of this clinical update on urticaria is to facilitate earlier diagnosis and appropriate treatment, minimise unnecessary investigations and facilitate early identification of those who might benefit from a specialist referral in response to and drawing from this revision. This should, in turn, lead to efficient, patient‐centred, cost‐effective care yielding improved outcomes for patients with urticaria.^25^ It is important to recognise that some interventions may be neither available nor affordable in all instances.

### 5.1 Methodology

A collaborative distillation of specialist guidelines by the guideline authors and knowledgeable primary care physicians: The guidelines referred to were contextualised and placed in the context of the non‐specialist to translate that guideline into a useful tool for non‐specialists.

## 6 URTICARIA DEFINITION

Urticaria is a condition characterised by the development of wheals (hives), angioedema, or both. It is further classified as acute (<6 weeks) or chronic (>6 weeks).

Wheals are superficial skin swellings of variable size, usually surrounded by reflex erythema, and they come with an itching or sometimes burning sensation. They are short lived, with the skin returning to its normal appearance, usually within 30 min to 24 h. (cf: photographs of wheals).

Angioedema is the sudden, pronounced erythematous or skin coloured swelling of the lower dermis and subcutis or mucous membranes. It is sometimes painful, rather than itchy, and its resolution is slower than that of wheals (can take up to 72 h). (cf: photographs of angioedema). It is to be noted that the angioedema occurring with urticaria is mast call mediated and does not result in fatalities: It tends to be rapid in onset. Bradykinin induced angioedema (hereditary angioedema, angiotensin converting enzyme inhibitor (ACE‐I) induced angioedema) differs in that there is often a slower evolution with the potential for fatality.^26^

Chronic Urticaria (CU) is further divided into two broad categories: CSU, and more rarely, chronic inducible urticaria (CIndU), which has clear precipitating factors (e.g. cold, heat, pressure, exercise etc).

### 6.1 Clinical vignette: (Hypothetical case)

A patient presented with acute urticaria subsequent to a viral infection in the early 2000's. Standard dose antihistamines proved ineffective, however symptoms were controlled with oral corticosteroids (OCS). In view of ongoing symptoms when steroids were withdrawn, the patient was referred to a dermatologist who performed multiple investigations. She was tried on varying combinations of antihistamines to no avail. Ciclosporin was effective but renal impairment became rapidly evident. Other treatments were tried, doxepin, methotrexate, mycophenolate mofetil and psoralen + ultraviolet light A, to no benefit. Some 10 years later she was referred to a dermatologist with a special interest in urticaria. A diagnosis of CSU was confirmed. The patient was weaned off OCS with a rapid increase of Urticaria Activity Score to nearly the maximum. Omalizumab was commenced, with rapid resolution of symptoms. After 6 months, omalizumab was discontinued with a return of symptoms. She now receives omalizumab regularly with a break every 6 months to determine whether spontaneous remission has occurred.

This vignette illustrates many of the pitfalls of CSU management: delay in confirming diagnosis, overexposure to oral steroids and inappropriate use of other medications.

There is a general perception by non‐specialists that urticaria, whether acute or chronic, is an allergic phenomenon, promoting a cascade of expensive and inappropriate investigations, the results of which may be misleading. Although urticaria is often thought to be due to type 1 hypersensitivity, (immunoglobulinE (IgE) mediated allergy), this is infrequently the case and very rarely without involvement of other body systems (cardio‐respiratory, gastrointestinal) and very rarely the case for chronic urticaria. Urticaria, may be precipitated by upper respiratory tract viral infections or the intake of non‐steroidal anti‐inflammatory drugs (NSAIDs), other medications or even foodstuffs.^27^ Identification of such triggers may be helpful in patient education and management.

Allergic acute urticaria must be distinguished from systemic anaphylactic reactions to allergens where wheals and/or angioedema occur together with extracutaneous signs and symptoms (Table 1 adapted from Muraro et al.^28^).

**TABLE 1:** Definition of anaphylaxis in the context of urticaria and/or angioedema

Acute onset of wheals with or without angioedema AND acute respiratory compromise (acute bronchoconstriction) and shock (hypotonia, collapse, syncope with or without incontinence)
Or:
Any TWO of: rapid onset wheals with or without angioedema
And/or respiratory compromise
And/or reduced BP with associated symptoms
And/or acute vomiting or severe crampy abdominal pain with or without diarrhea
And/or reduced blood pressure after exposure to known allergen.

A retrospective study of anaphylaxis presenting to ED revealed that two‐thirds of anaphylaxis patients (67.1%) presented with wheals/urticaria and 41.3% with angioedema.^29^ The distinction between the disease urticaria and wheals in anaphylaxis can only be made by looking at the time course. Wheals in anaphylaxis fade after a short period of at most a few hours whereas in acute urticaria although the majority disappear within 2–3 days, occasionally they may persist for up to 21 days.^30^ It is critical to understand that anaphylaxis with wheals is NOT acute urticaria and equally acute urticaria does not equate to anaphylaxis.

### 6.2 Natural history of acute urticaria

Acute urticaria, like all urticaria, can present with wheals, angioedema, or both. Most episodes of acute urticaria, independent of symptoms, resolve within 2–3 days. Single wheals usually resolve in 24 h but may recur in a different location, Suggest replace but with however but angioedema may take up to 72 h to resolve.^31^ In the absence of systemic symptoms, reassurance is of great importance to the patient or the parents of the patient.

Patients with inducible urticaria often come to their GP after first experiencing wheals in response to a physical trigger: cold, heat, pressure, water UV light etc. To date there is limited data on the natural course of inducible urticarias. Like in CSU, most patients are expected to experience remission after several years of having the disease.

### 6.3 Natural history of CSU

Most cases of acute urticaria are acute spontaneous urticaria, rather than acute inducible urticaria. Independent of symptoms, acute urticaria usually resolves within 2–3 days and by week 6 the latest.

The natural history of CSU is one of resolution, which when it occurs, occurs rapidly: some 50% will resolve by 6 months after diagnosis, 30% at 3 years 10% at 10 years with some 8% suffering for more than 25 years.^32^ In European populations most patients suffer from CSU for more than 1 year with a considerable number of patients still affected for longer than 5 years.^7^

### 6.4 Natural history of CIndU

CIndU, a subset of CU, lasts for several years in most patients before it shows spontaneous remission.^33^ Until spontaneous remission occurs, many patients are severely impaired in their quality of life. Many patients go to great lengths to avoid triggers of whealing which comes with a high quality of life burden and many restrictions. A thorough history and provocation testing are essential for establishing the diagnosis of CIndU as there still no biomarkers to help with this.

### 6.5 Impact of management of CU

Approximately 40% of patients with CSU will achieve disease control with high dose (4x standard dose) second‐generation oral antihistamines: this further increases up to 70% when using omalizumab with very much smaller incremental benefits gained from addition of other treatments (leukotriene receptor antagonists (LTRAs), ciclosporin etc, which should be administered only by specialists).

Inducible urticarias are more difficult to treat than spontaneous despite the fact that patients with some forms of CIndU can avoid precipitating factors to some extent; exposure to cold, heat, UV radiation, pressure for example.^34^

### 6.6 What are the challenges presented by the management of urticaria?

Currently, in common with allergy^35^ there are no clearly defined health care pathways for patients suffering from urticaria resulting in anxiety and frustration from patients who felt that there was great expenditure in time and resources before they received a diagnosis.^7^

From the investigations which are requested there appears to be a belief by both clinicians and patients that urticaria is an allergic phenomenon, with allergy investigations performed in more than half patients seen, along with many other irrelevant investigations including serology for infections or antibody profiling: however, this is as true for specialists as for GPs.^36^ There is also evidence that patients with urticaria are often initially misdiagnosed as having allergy; In an Irish series of 100 consecutive referrals to a specialist allergy service, 71% were referred with suspected food allergy as the main concern; in 61% wheals and/or angioedema was their presenting symptom and 56% received the final diagnosis of CSU. Only 9% had IgE‐mediated food allergy, the majority of whom presented with anaphylaxis.^17^

Many patients with CSU are also likely to be undertreated by both dosing and type of medication with many patients receiving first generation antihistamines even though these provide inferior outcomes and greater side effects.^37^ Second generation, non‐sedating antihistamines should be used at up to four times standard dose in those patients with insufficient response to standard dose, although this remains off‐licence, but is endorsed by guidelines and supported by substantial evidence.^24^ ^,^ ^38^ Equally, after an initial flurry of activity it would appear that for many patients the time from first presentation to receiving a specialist opinion is inordinately great, averaging 4 years.^7^ This in turn results in considerable health care resource utilisation.^39^

In addition, there is a problem with angioedema. We know that patients with hereditary angioedema (HAE) also experience long delays in diagnosis and high rates of misdiagnosis. The most common misdiagnoses are “allergic angioedema” and appendicitis. Allergic angioedema (and HAE) is often suspected by GPs in patients with CSU who present with recurrent angioedema with no wheals.^13^

### 6.7 How should acute urticaria be managed in primary care and in the ED?

#### 6.7.1 Clinical history

**FIGURE 1:**
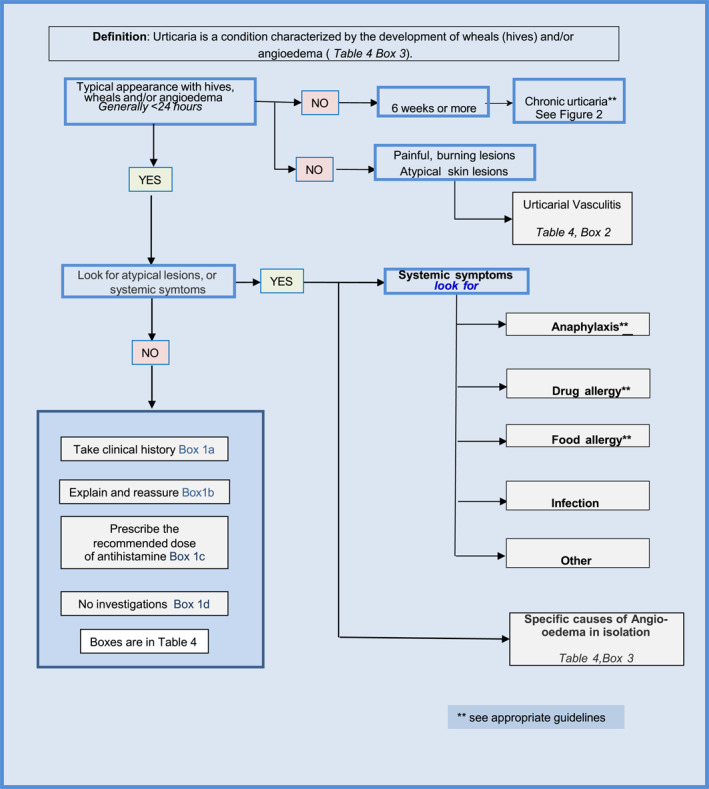
Pictogram describing diagnostic approach to acute urticaria in primary care: more information be found by referring to the boxes in Table 4

The presentation of the patient may occur during the event or after the event has resolved (Figure 1). In either case, the clinical history is vital in attempting to both make the initial diagnosis and to assess the aetiological factors involved. Frequently, the diagnosis is self‐evident if the rash is present at time of presentation but equally many patients present after the event so history should include a time‐line of the index event, how long the rash lasted and whether any photos were taken (particularly in this COVID era, many first contacts may be made remotely and the use of photos taken on mobile phones at the earliest possible time is to be recommended). Were there any factors such as a current respiratory viral infection or the use of NSAIDs or other drugs? ACE‐inhibitor related disease presents as angio‐oedema rather than acute urticaria.^40^ Were there any systemic associated features such as vomiting, diarrhoea, vaso‐vagal reaction or difficulty in breathing, suggesting a diagnosis of anaphylaxis. Ask if they can induce their urticaria and how long does it last for. Was there exposure to cold, pressure, UV light, sweat‐inducing activities/situations or other triggers of inducible urticaria?

## 7 FOCUSSED HISTORY, TAKING THE FOLLOWING ITEMS INTO CONSIDERATION (ADAPTED FROM REFERENCE Ferrer et al.^41^)

1. Time of onset of disease
2. Shape, size, severity, frequency/duration and distribution of wheals
3. Location, frequency and duration of angioedema
4. Associated symptoms, for example, bone/joint pain, fever, abdominal cramps
5. Family and personal history regarding wheals and angioedema
6. Induction by physical triggers or sweat‐including activities/situations (e.g. exercise)
7. Occurrence in relation to daytime, weekends, menstrual cycle, holidays, and foreign travel
8. Occurrence in relation to foods or drugs (e.g. NSAIDs, ACE‐Inhibitors)
9. Occurrence in relation to infections, stress
10. Previous or current allergies, infections, internal/autoimmune diseases, gastric/intestinal problems or other disorders
11. Social and occupational history, leisure activities
12. Previous diagnostic procedures/results
13. Previous therapy and response to therapy including dosage and duration
14. Full skin examination

Furthermore, given the anxiety and/or depression which frequently accompanies CU it is important that such co‐morbidities are actively sought in the consultation.^42^

## 8 DIAGNOSTIC APPROACH

The diagnostic approach should include (Figure 1):

- confirming the diagnosis, consider differential diagnosis and medication history and include hypersensitivity reactions to insect bites which may resemble urticaria (previously called “papular urticaria”)
- Ruling out anaphylaxis (cf Table 1) (in case of anaphylaxis, perform safety netting ‐> administering adrenalin, being provided with an emergency plan (including adrenalin auto injector) and arranging follow up.^43^) If anaphylaxis is suspected, although not generally available in primary care, the taking of serial serum tryptase levels may assist in arriving at the diagnosis.
- Rule out drug allergy (usually possible by history alone).^44^
- Rule out presentations of other dermatological disorders.

If the clinical diagnosis is confirmed, performing further investigations is the exception not the rule. Patients should not be tested for underlying causes, unless clues from the history point to an allergic reaction.^45^ Unnecessary investigations are not only unhelpful but may throw up confounding results.^46^, ^47^

In the absence of systemic symptoms, reassurance is of great importance to the patient or the parents of the patient as the rash and especially angioedema may appear very threatening causing fearfulness of further progression to anaphylaxis. Patients should be told: that generally the causes of acute urticaria are unknown although stress and infection can be contributing factors and, importantly, require no investigations. Specifically, cold induced urticaria is potentially serious and caution about swimming in cold water and eating ice cream should be given before expert review occurs.

Referral at this stage should be considered for suspected drug reaction (confirm or refute penicillin allergy) or anaphylaxis if this is considered a possibility. Elucidating the role of NSAID hypersensitivity is very complex and may require specialist assessment.^48^ Any drug suspected of being a trigger factor should be withheld until after specialist review.

Other factors which should prompt referral are suspicion of urticarial vasculitis (typically skin lesions are painful rather than itchy), autoimmune disease, or if the patient has atypical symptoms such as bone pain or is systemically unwell.

Referral should also be considered in patients with angioedema in isolation for investigation of bradykinin‐mediated angioedema.

## 9 EXAMINATION

The whole of the skin should be examined to detect whether any lesions are present and whether their appearance is that of urticaria/angioedema. A photographic record retained in the patient's electronic medical record may be useful. Also look for colour of the lesions, skin pain and systemic signs or symptoms. Given the association with thyroid disease it is worthwhile checking for the presence of a goitre.

## 10 PHOTOS OF WHEALS/URTICARIAL RASH

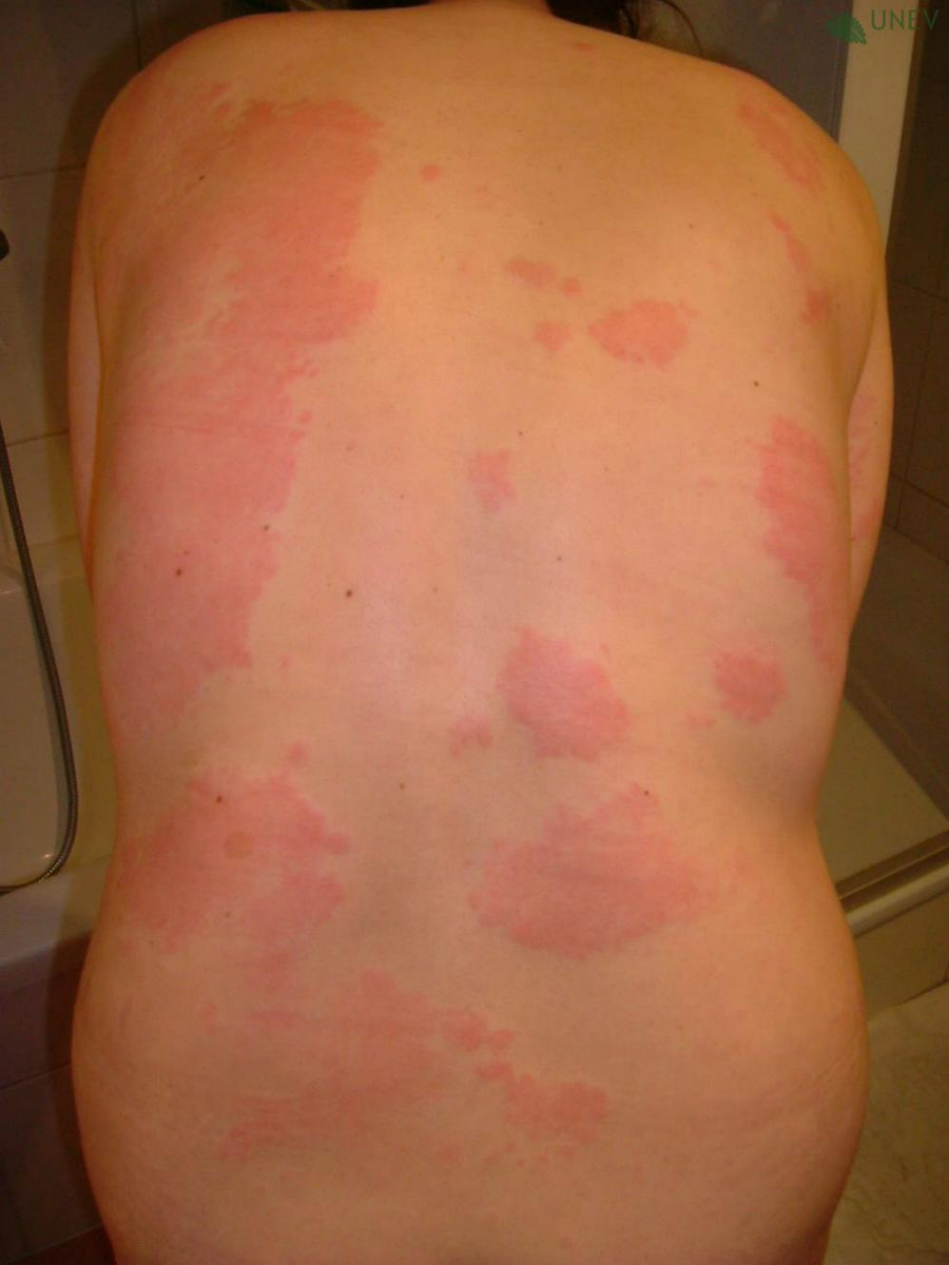

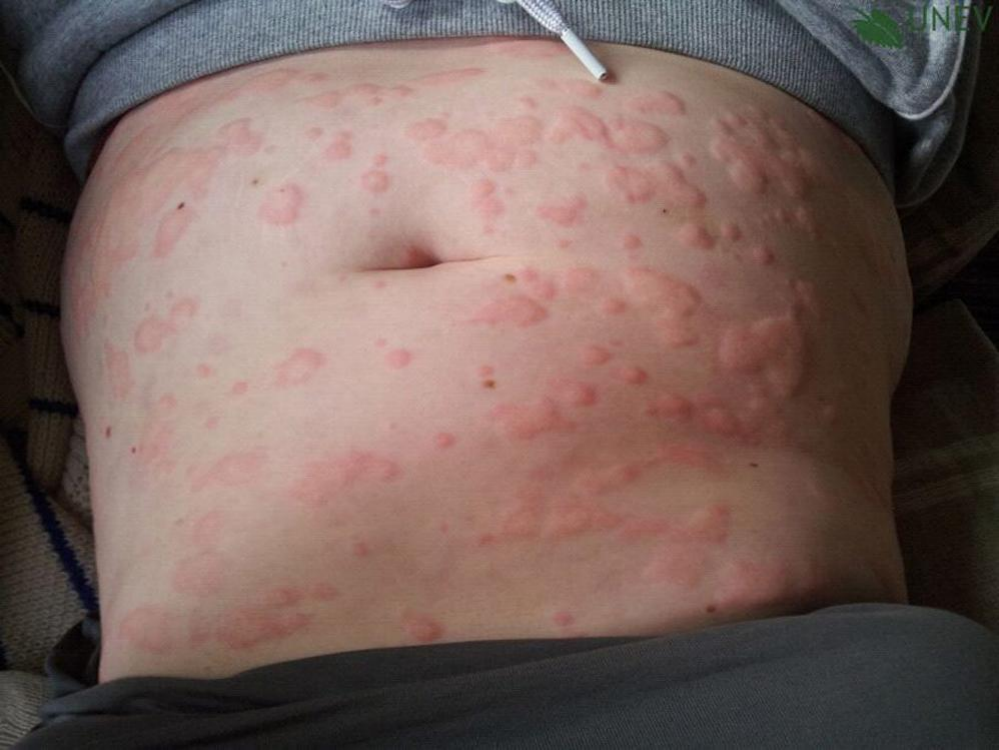

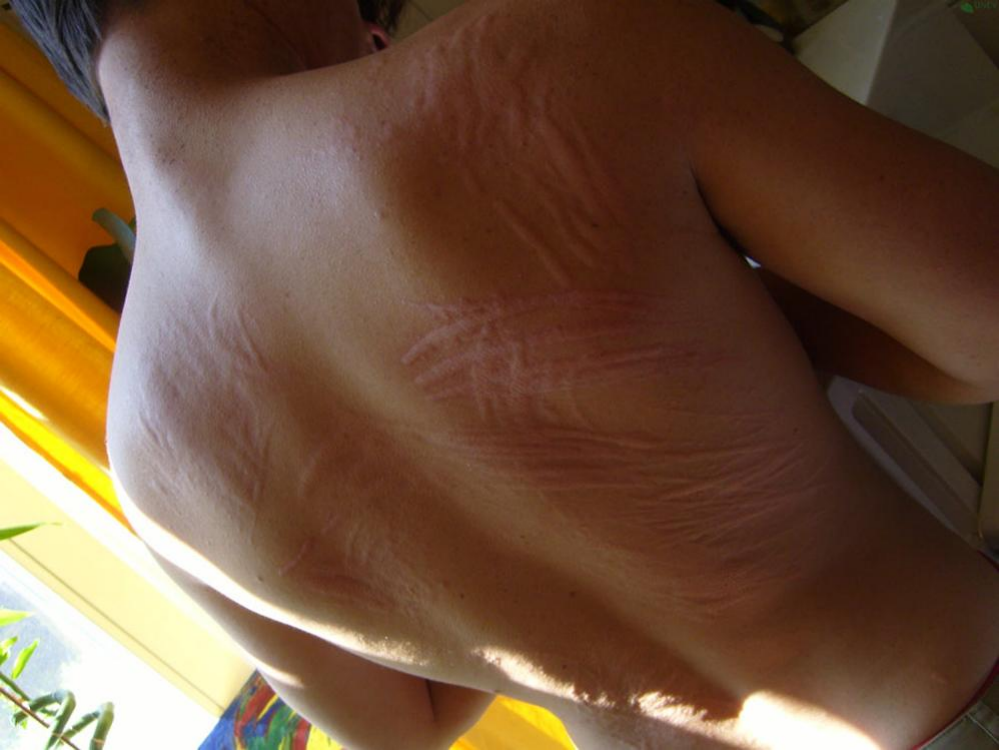

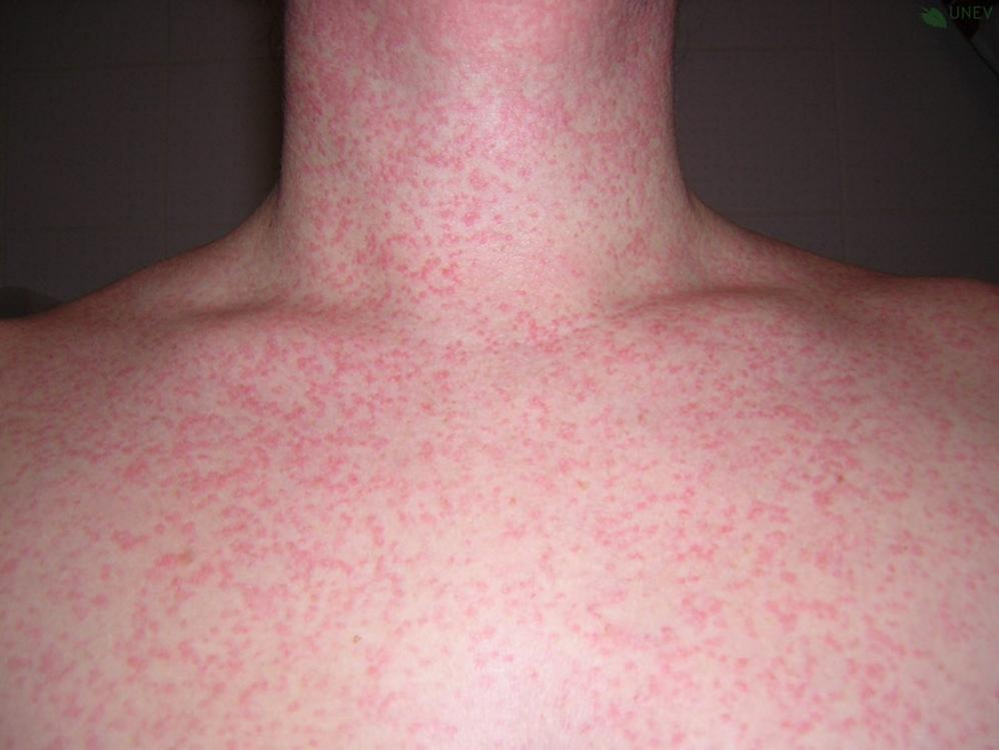

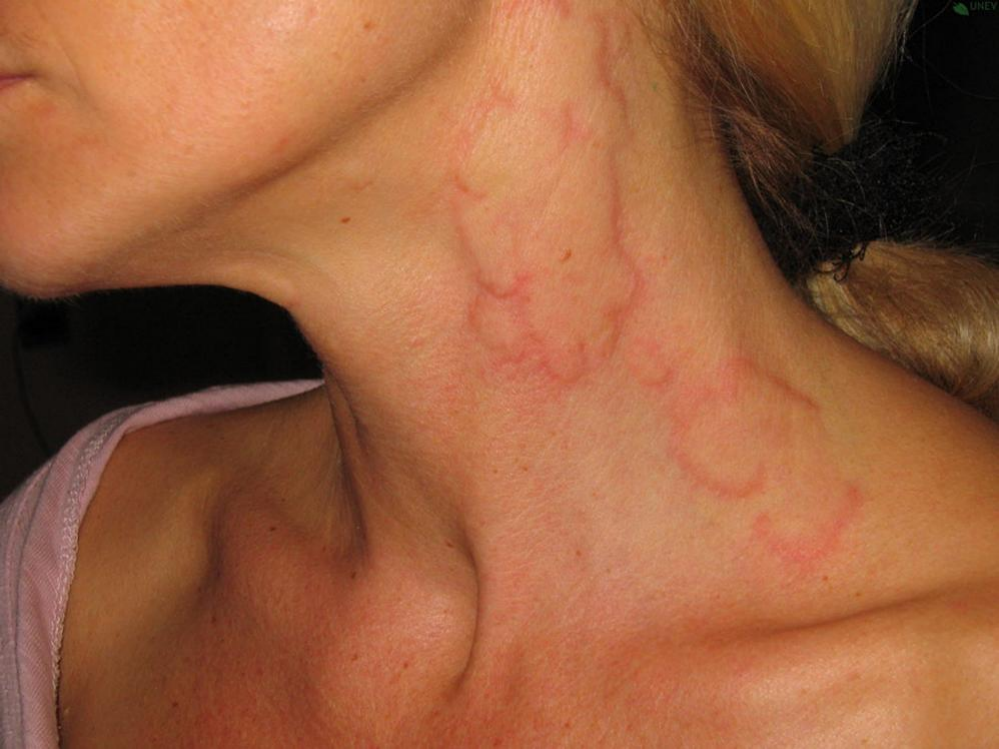

## 11 PHOTOS OF ANGIOEDEMA

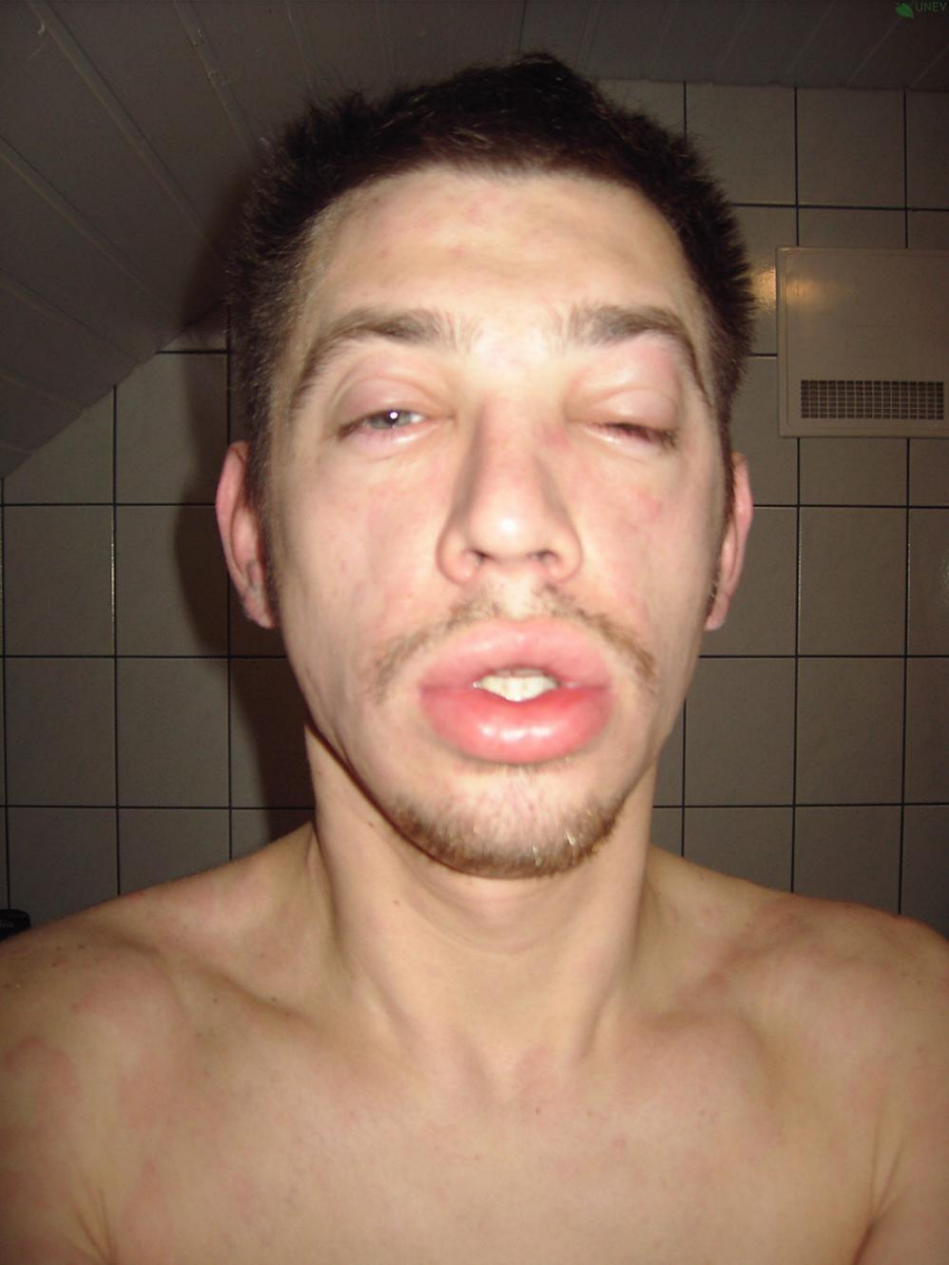

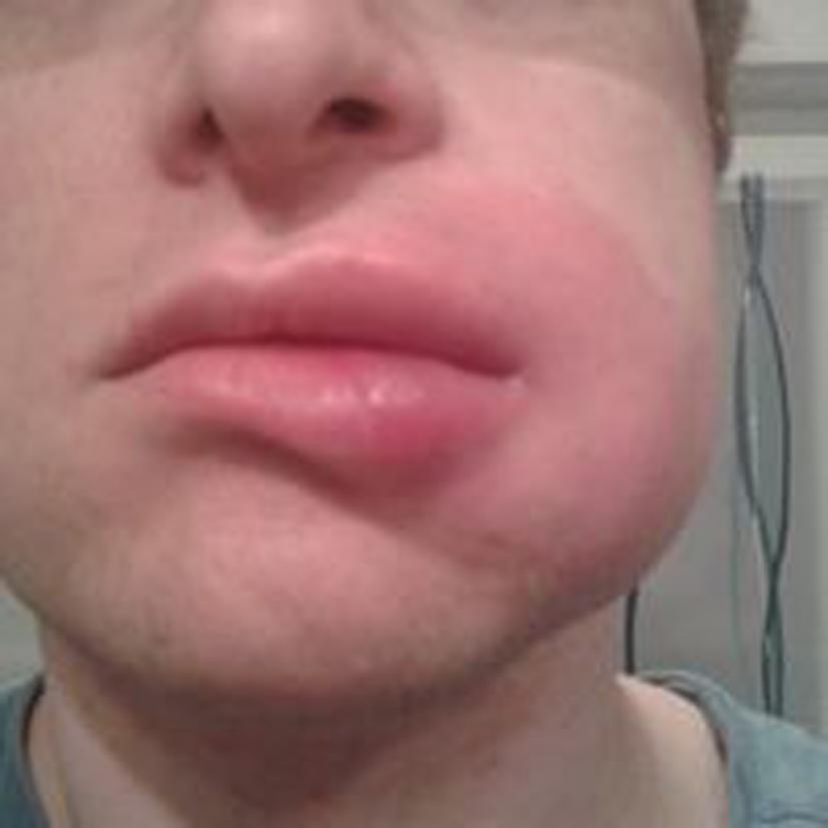

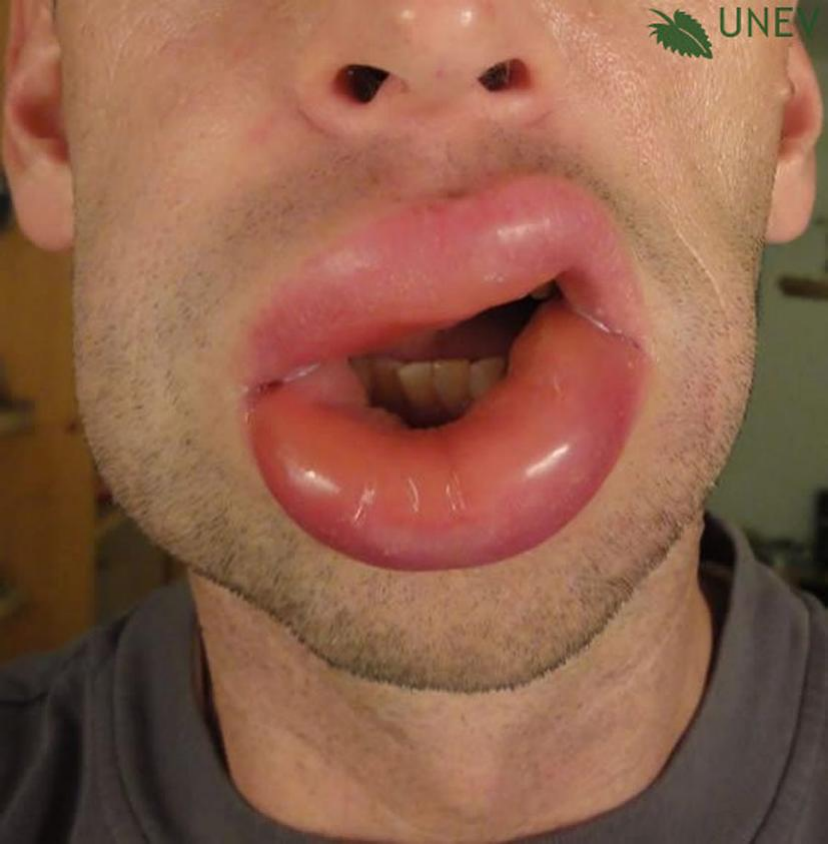

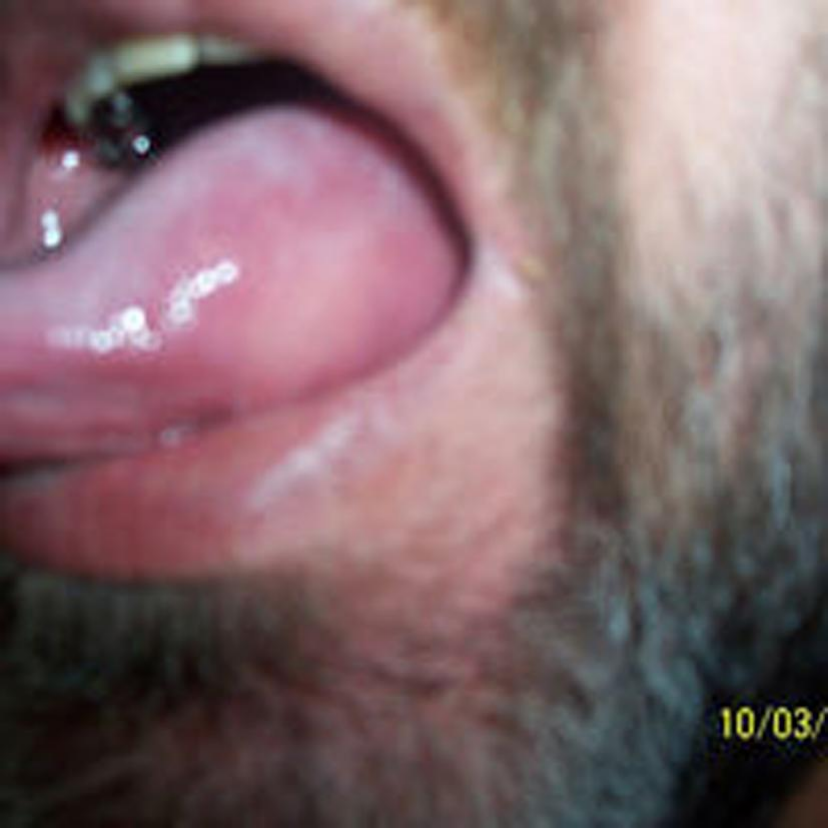

**Urticaria and angioedema photos used with permission from:** https://www.urtikaria.net/de/fuer‐betroffene/galerie/album‐urtikaria.html, who hold patient consent to use their photographs for publication and education. Further photographs are available by clicking in the link above (text in German).

## 12 MANAGEMENT OF URTICARIA

### 12.1 Management of acute urticaria

The aim of treatment is complete symptom control achieved through reassurance, education and judicious use of medication.

Arguably the single most important factor in management is the giving of explanation of what is happening/has happened and reassurance concerning the long‐term outlook (Cf Table 2), which is why a broad understanding of the natural history of urticaria is important. This action defuses fear and anxiety by demonstrating that there is a large degree of certainty about the outcome. Fixed beliefs about allergy will need to be holistically explained with reference to food diaries or guidelines; modification of lifestyle with less stress and better wellbeing could also be advocated. This is an area which has not yet been researched for acute urticaria.^25^

**TABLE 2:** How should primary care physicians counsel patients with acute urticaria?

Key phrases in consultation
• I know what this is.
• This is not dangerous.
• This is not an allergy.
• This will eventually go away by itself.
• Investigations are not helpful
• This can be easily treated.
• What are your fears?

Reassurance acknowledges that acute urticaria is frightening and can cause transient problems with sleep disturbance and body image but is not dangerous; the cause of acute urticaria is rarely precisely identified: that the majority resolve within 3–5 days: that if it persists there is still no indication to refer until 6 weeks have elapsed, unless symptoms are deteriorating or becoming more severe. That at 6 weeks, limited blood tests will be performed (full blood count and differential, C‐reactive protein [CRP] or erythrocyte sedimentation rate [ESR]) coupled with increasing the dose of second generation antihistamines up to fourfold standard dose accompanied by monitoring disease activity and control with easy to use tools such as the urticaria activity score (UAS)^55^ or urticaria control test (UCT).^49^ If remission is achieved referral is not necessary but if remission is not achieved then the patient should be referred for further investigations which may not necessarily reveal the precise cause of their now chronic urticaria, but – together with the use of more effective treatments ‐ should ultimately result in resolution of symptoms. That in virtually all cases, at some time, spontaneous remission will occur, and that disease activity will be controlled by continuing medication until this occurs. It is important to listen empathically sharing management plans with the patient.

### 12.2 Investigations

If the diagnosis reached is acute spontaneous urticaria there is no indication for investigations of any kind. This needs to be communicated transparently to the patient who often seeks investigations to determine what has caused their distressing symptoms and documented in the notes.

If the urticaria progresses and becomes chronic spontaneous urticaria it is worthwhile, at this time, performing a full blood count and differential and either CRP, ESR, so that these can be included in the referral letter.

### 12.3 Pharmacotherapy of acute spontaneous urticaria

Urticaria is a phenomenon largely driven by the release of histamine, thus the logical approach is to treat the disorder with standard dose of long‐acting non‐sedating antihistamine.

The patient should be told that apart from antihistamines there is no other effective treatment available in primary care: oral steroids are rarely indicated, due to their side effects: even short courses of oral steroids in otherwise healthy individuals are associated with a bewildering array of adverse events.^50^ Longer term use, studied in other chronic diseases similarly reveal the high frequency and severity of side effects.^51^ By and large, treatment should be continued until the rash disappears and may then be discontinued. The patient should be asked to report back if symptoms deteriorate or persist beyond 6 weeks when a modified approach to management is adopted. If symptoms are of 6 weeks or more duration, by definition, the patient has CU.

If patients (or parents) are very distressed, a very short burst of oral steroids could be given for 3–5 days at no higher than 1 mg per Kg bodyweight daily, but it is important that the recipient is fully appraised of the potential risk benefit ratio.^50^

Treatment should commence with a standard‐dose second generation antihistamine.^38^ This means age and weight adjusted (and unlicensed) dosing in children^24^ The majority of cases of acute spontaneous urticaria will resolve within a week, but some will last longer, particularly those with hypersensitivity reaction to insect bites, which may last a for some weeks, especially if the subject continues to be bitten/stung.

The use of first‐generation antihistamines is discouraged in all age groups due to their poor side effect profile.^52^

Note: There is no role in primary care for mixing different antihistamines nor for adding H2 blockers (cimetidine, nizatidine etc) unless recommended in local guidelines which may have been formulated adapted to local availability of medications. Similarly, there is no role for leukotriene receptor antagonists (e.g. montelukast), although there are differences in opinion between Europe and North America.^53^

### 12.4 Diagnosis and management of CU in primary care

**FIGURE 2:**
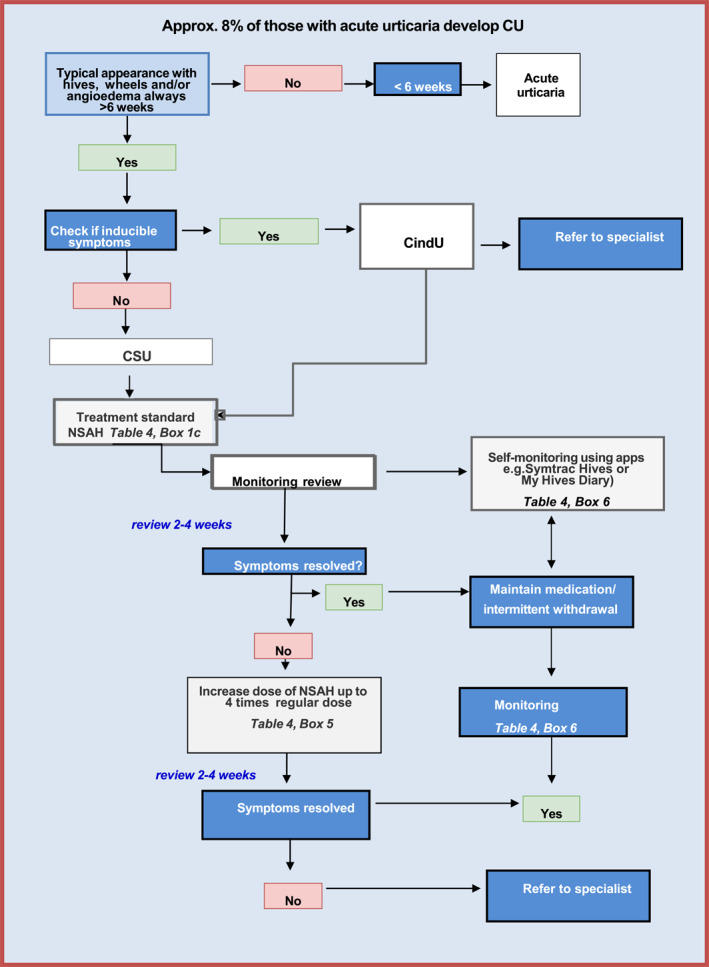
Diagnosis and management of CU in primary care: Further information may be found in the boxes in Table 4

CU occurs when symptoms have continued to occur for 6 weeks (Figure 2).

If symptoms are controlled on any dose of a long‐acting non‐sedating antihistamine up to a maximum of four times the standard dose or if the symptoms have spontaneously remitted, no referral is necessary. Note: it may take 1–2 weeks before symptoms come under control. It is important to impress on the patient of taking the medication daily in order to retain remission or symptoms will relapse. However, when remission is achieved, therapy should be withdrawn every 3–6 months to discover whether natural remission has occurred.

The corollary of this is that all patients who remain symptomatic and who have been adherent to medication, should be referred for specialist evaluation. CU is a complex disease area, suggesting that the referral should be to a person with extensive knowledge of this disorder in order to achieve the best outcome.^54^

## 13 REFERRAL

If urticaria is thought to be associated with a drug reaction, food allergy or anaphylaxis, urgent referral should be made to an allergist or dermatologist without undue delay.

By definition, acute urticaria is a self‐limiting disorder with a duration of less than 6 weeks which will not benefit from further investigation or referral.

Referral should be undertaken if the patient has had symptoms for six or more weeks in spite of an adequate trial of high dose second generation antihistamine therapy (four times standard dose) or if there is any diagnostic uncertainty for example, urticarial vasculitis. The rationale for referral should be explained to and discussed with the patient (Table 3).

**TABLE 3:** What should primary care physicians tell patients when they refer to a specialist?

• They may consider additional special tests.
• They have a range of different treatment options.
• They have a greater chance of achieving control.

The specialist may perform further investigations with three aims in mind (1) excluding differential diagnoses, (2) assessment of disease activity, impact and disease control and (3) to identify triggers or if suspected, underlying causes.^19^

The primary care physician or ED clinician does not need to understand the complexity of investigations which may be performed by those who are expert in this area. However, they should have a broad appreciation of what will happen next and be able to explain it. (see below). They should also perform a Full Blood Count and ESR or CRP to be included in the referral.

### 13.1 Diagnostic approach to CU in specialist care

The details of the events to date with a clear description of the rash and timings and therapy tried will be detailed in the referral letter along with any photos if possible. This will be accompanied by the results of the simple blood tests performed mentioned above. The patient will attend with photographs taken during events, as well as documentation of disease activity and control by use of the Urticaria Activity Score 7 (UAS7),^55^ UCT (Urticaria Control Test)^49^ or both.

It is likely that a more detailed history will be taken in the search for any precipitating triggers, facilitating conditions and comorbidities. Based on the clues from the detailed history, additional investigations may be performed, especially in patients with longstanding and severe disease. There are a variety of provocation tests and blood tests which may be performed by the specialist in order to determine if there is a treatable condition that contributes to a patient's urticaria. In many patients, no such precipitating factors are identified. However, even in specialist care there is a clear need to be judicious in the use of investigations.^47^

If CIndU, in which urticaria develops in response to clearly defined stimuli such as cold, vibration, radiation, heat, water etc is suspected, provocation testing should precede treatment. Trigger threshold testing is useful, but availability of threshold testing is often limited to specialist centres.^56^ However simple, largely asymptomatic inducible urticaria such as mild cases of symptomatic dermographism or pressure urticaria can be managed in primary care with explanation, reassurance, and antihistamine treatment. Omalizumab is not licensed for the use in CIndU and therefore not used as often as in CSU.

Specialists will likely confirm that antihistamines at up to four times the standard are ineffective before progressing to other treatment options. Adherence to antihistamines and the effects on signs and symptoms will be documented, often with the help of the urticaria activity score and/or the angioedema activity score.

These tools will also be used to monitor and optimize the therapy with third line treatment options such as omalizumab and/or cyclosporin.

## 14 CONCLUSIONS: SUMMARY, UNMET NEEDS AND OPEN QUESTIONS

In summary, urticaria is relatively simple to manage if recognised and treatment started in partnership with the patient. Reassurance, shared decision making and simple standard treatments are the bedrock of management. For CU one of the challenges is to get the patient to the specialist in an appropriate time frame in order to confirm the diagnosis and relieve suffering if the patient is unresponsive to treatments easily provided in primary care; a major need is the construction of a simple value based care pathway ending at a facility/clinic which can deal effectively with this disease.^57^ But an equally pressing need is to provide primary care with the necessary knowledge and skills to manage this common condition.^11^ There is also an urgent need for a large scale population based study of the true prevalence and cumulative prevalence of urticaria, preferably using a longitudinal data base from primary care in an attempt to assess the unmet needs of patients with urticaria such as has been performed in other disease areas.^58^

There is a clear need to resist the temptation for any unnecessary investigations which, although well intentioned, reveal nothing and may be counterproductive by increasing uncertainty.

There is a need to determine at what frequency intervals biologic treatment for CSU should be withdrawn in order to determine whether spontaneous resolution has occurred which will facilitate treatment withdrawal.^32^, ^59^, ^60^

Please note: Figures 1 and 2, and Table 4 are adapted from Ryan^61^: Ryan D, Flokstra‐de Blok B, Clark E, Gaudin C, Mamodaly M, Kocks J, van der Velde J, Angier E, Romberg K, Gawlik R, Demoly P. Allergic and hypersensitivity conditions in non‐specialist care: flow‐diagrams to support clinical practice 19 March 2022: https://doi.org/10.1111/all.15273.

**TABLE 4:** Boxes containing further details

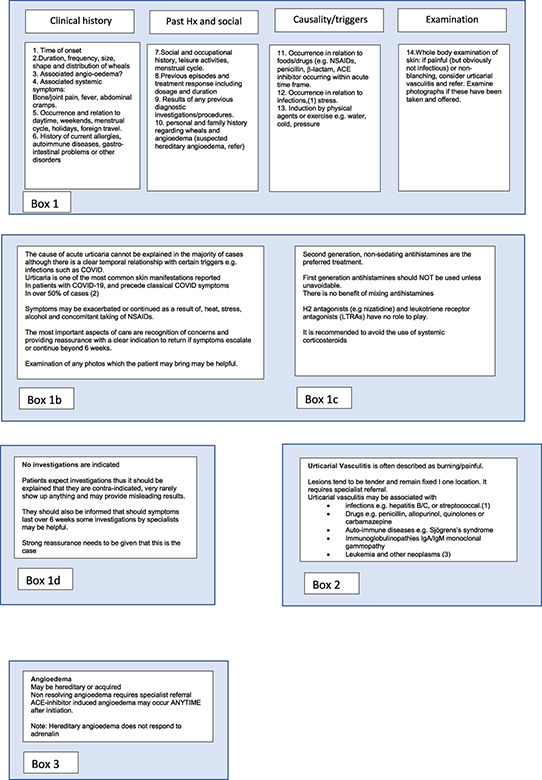
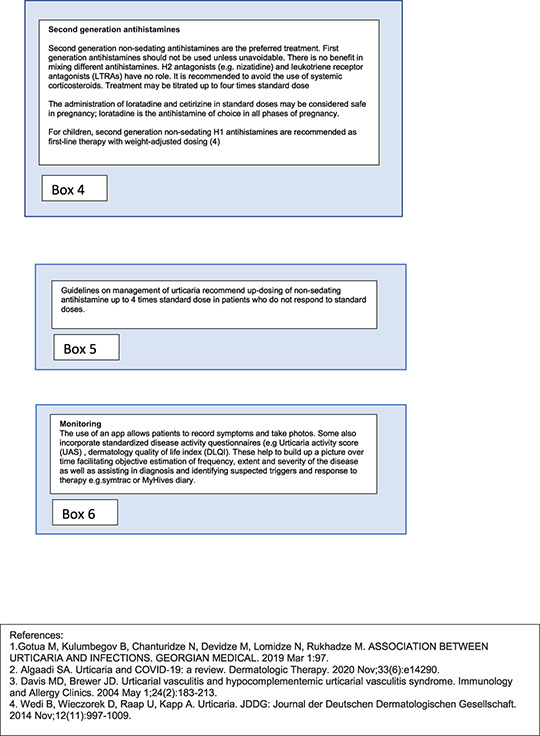

## AUTHOR CONTRIBUTIONS

**Dermot Ryan**: Conceptualization (Lead); Data curation (Equal); Formal analysis (Equal); Methodology (Equal); Project administration (Lead); Writing – original draft (Lead); Writing – review & editing (Lead). **Luciana K. Tanno**: Data curation (Equal); Formal analysis (Equal); Writing – original draft (Equal); Writing – review & editing (Equal). **Elizabeth Angier**: Writing – original draft (Supporting); Writing – review & editing (Supporting). **Evangeline Clark**: Resources (Supporting); Writing – review & editing (Supporting). **David Price**: Writing – original draft (Equal); Writing – review & editing (Equal). **Torsten Zuberbier**: Conceptualization (Equal); Formal analysis (Equal); Methodology (Equal); Writing – review & editing (Equal). **Marcus Maurer**: Conceptualization (Equal); Formal analysis (Equal); Methodology (Equal); Project administration (Equal); Writing – original draft (Equal); Writing – review & editing (Equal).

## CONFLICTS OF INTEREST

David Price has advisory board membership with Amgen, AstraZeneca, Boehringer Ingelheim, Chiesi, Circassia, Viatris, Mundipharma, Novartis, Regeneron Pharmaceuticals, Sanofi Genzyme, Teva Pharmaceuticals and Thermofisher; consultancy agreements with Amgen, AstraZeneca, Boehringer Ingelheim, Chiesi, GlaxoSmithKline, Viatris, Mundipharma, Novartis, Pfizer, Teva Pharmaceuticals and Theravance; grants and unrestricted funding for investigator‐initiated studies (conducted through Observational and Pragmatic Research Institute Pte Ltd) from AstraZeneca, Boehringer Ingelheim, Chiesi, Circassia, Viatris, Mundipharma, Novartis, Pfizer, Regeneron Pharmaceuticals, Sanofi Genzyme, Teva Pharmaceuticals, Theravance and UK National Health Service; payment for lectures/speaking engagements from AstraZeneca, Boehringer Ingelheim, Chiesi, Cipla, GlaxoSmithKline, Viatris, Mundipharma, Novartis, Pfizer, Regeneron Pharmaceuticals, Sanofi Genzyme and Teva Pharmaceuticals; payment for travel/accommodation/meeting expenses from AstraZeneca, Boehringer Ingelheim, Circassia, Mundipharma, Novartis, Teva Pharmaceuticals and Thermofisher; funding for patient enrolment or completion of research from Novartis; stock/stock options from AKL Research and Development Ltd which produces phytopharmaceuticals; owns 74% of the social enterprise Optimum Patient Care Ltd (Australia and UK) and 74% of Observational and Pragmatic Research Institute Pte Ltd (Singapore); 5% shareholding in Timestamp which develops adherence monitoring technology; is peer reviewer for grant committees of the UK Efficacy and Mechanism Evaluation programme, and Health Technology Assessment; and was an expert witness for GlaxoSmithKline. Marcus Maurer declares no COI relevant to this MS. Outside of it, he is or recently was a speaker and/or advisor for and/or has received research funding from Allakos, Alnylam, Amgen, Aquestive, Aralez, Astria, ArgenX, AstraZeneca, BioCryst, Blueprint, Celldex, Centogene, CSL Behring, Dyax, FAES, Genentech, GIInnovation, GSK, Innate Pharma, Kalvista, Kyowa Kirin, Leo Pharma, Lilly, Menarini, Moxie, Novartis, Pfizer, Pharming, Pharvaris, Roche, Sanofi/Regeneron, Shire/Takeda, ThirdHarmonicBio, UCB, and Uriach. Dermot Ryan, Torsten Zuberbier, Elisabeth Angier, Luciana Tanno and Evangéline Clark declare no COI relevant to this MS.
